# Transcriptomic Analysis Revealed the Common and Divergent Responses of Maize Seedling Leaves to Cold and Heat Stresses

**DOI:** 10.3390/genes11080881

**Published:** 2020-08-03

**Authors:** Yongsheng Li, Xingrong Wang, Yue Li, Yanjun Zhang, Zuowang Gou, Xusheng Qi, Jinlin Zhang

**Affiliations:** 1 Key Laboratory of Grassland Livestock Industry Innovation, Ministry of Agriculture and Rural Affairs, State Key Laboratory of Grassland Agro-ecosystems, College of Pastoral Agriculture Science and Technology, Lanzhou University, Lanzhou 730020, China; 2 Crop Research Institute, Gansu Academy of Agricultural Sciences, Lanzhou 730070, China; wangxrzws@gsagr.ac.cn (X.W.); liyue@gsagr.ac.cn (Y.L.); zhangyanjun@gsagr.ac.cn (Y.Z.); gouzuowang@gsagr.ac.cn (Z.G.)

**Keywords:** maize, temperature stresses, transcription factors, hormone signaling, heat shock proteins, co-expression network

## Abstract

Temperature stresses (TS), including cold and heat stress, adversely affect the growth, development, and yield of maize (*Zea mays* L.). To clarify the molecular mechanisms of the tolerance of maize seedling leaves to TS, we applied transcriptomic sequencing of an inbred maize line, B73, with seedlings exposed to various temperature conditions, including normal temperature (NT, 25 °C), cold (4, 10, and 16 °C), and heat (37, 42, and 48 °C) stresses. Differentially expressed genes (DEGs) were detected in different comparison between the NT sample and each temperature-stressed sample, with 5358, 5485, 5312, 1095, 2006, and 4760 DEGs responding to TS of 4, 10, 16, 37, 42, and 48 °C, respectively. For cold and heat stresses, 189 DEGs enriched in the hydrogen peroxidase metabolic process, cellular modified amino acid metabolic process, and sulfur compound metabolic process were common. The DEGs encoding calcium signaling and reactive oxygen species scavenging enzymes demonstrated similar expression characterizations, whereas the DEGs encoding transcription factors, such as ERF, ARF, and HSF, hormone signaling, and heat shock proteins, displayed divergent expression models, implying both common and divergent responses to cold and heat stresses in maize seedling leaves. Co-expression network analysis showed that functional DEGs associated with the core regulators in response to cold and heat stresses were significantly correlated with TS, indicating their vital roles in cold and heat adaptation, respectively. Our investigation focused on the response to gradient TS, and the results presented a relatively comprehensive category of genes involved in differential TS responses. These will contribute a better understanding of the molecular mechanisms of maize seedling leaf responses to TS and provide valuable genetic resources for breeding TS tolerant varieties of maize.

## 1. Introduction

Maize, as a critical source of food, fuel, feed, and fibers, is one of the leading crops worldwide, originating from the Mexican highlands center and having diffused to low temperature regions of temperate climates [1]. The adaptation to temperature gradients is the most important determinant for maize to grow in worldwide regions. Although maize originated in tropical regions, heat stress also causes adverse effects on maize, such as changed leaf morphology; reduced CO_2_ assimilation; effects on the flowering time, pollination, and silking; and decreased kernel yield [2,3]. Maize also faces challenges in cultivation in temperate regions, as it is highly sensitive to low temperatures [4]. Cold stresses of maize could damage the photosynthetic system, reduce the enzyme activity of photosynthesis, affect the carbohydrate status of the leaves, modify the cell wall, and cause water deficits [5,6,7,8,9,10]. These changes of phenotype when experiencing temperature stresses (TS) occurred through accurate regulation of the gene expression and are genetically controlled. Screening the candidate genes contributing to heat stress at the molecular level is, thus, possible. Dissection of the stress response genes that are associated with TS could help identify vital regulators and pathways as potential targets for breeding tolerant varieties adaptable to fluctuating temperature environments.

Recently, scientists have made significant progress in understanding the mechanisms of TS perception and signaling [11,12]. Under low-temperature, the cytosolic Ca^2+^ levels of plant cells are increased through Ca^2+^ channels, which will induce Ca^2+^ signatures to induce downstream gene expression, such as C-repeat binding factors (CBFs) [13,14]. CBFs have been identified as the core regulators activating the cold-response gene expression in *Arabidopsis* [15,16]. Dehydration-responsive elements binding factors (DREBs) belong to this group of regulators. A total of three CBFs were identified in *Arabidopsis* and rapidly induced by low temperatures [17], which were positively regulated by the two bHLH transcription factors, *ICE1* and *ICE2* [18,19]. The expression of CBFs was also negatively regulated by *MYB15* and *EIN3* in *Arabidopsis* [20,21]. In addition, the CBF-dependent pathway involved in the cold response, a novel pathway called tolerant to chilling and freezing 1 (*TCF1*), was identified and involved in regulating cold responses through a CBF-independent pathway [22].

The investigations in heat stress also demonstrated that Ca^2+^ is the initial and indispensable factor involved in heat stress responses [23,24,25,26]. In the transcription factor (TF) family, heat response factors (HSFs) are the master regulators necessary for the activation of the transcriptional networks in the heat stress response [12]. The mutation of *HsfA1s* in *Arabidopsis* and tomato reduced the induction of heat stress response genes [27,28,29], and researchers predicted that they may directly regulate the expression of important heat stress-responsive transcription factors, including *DREB2A*, *HsfA2*, and *HsfBs* [29]. A knockdown mutation of *HsfA3* led to reduced expression of heat shock proteins (HSP) genes during heat stress [30]. Other TFs, such as *NAC109* and *bZIP28*, were also important in regulating heat stress responses through a *HsfA1s*-independent pathway [31,32]. These studies imply that Ca^2+^ signaling and TFs mediated pathways play vital roles in TS responses.

Maize is sensitive to heat and cold stresses, and some studies have focused on the response to TS in maize, including the comparative transcriptome analysis of different genotypes with differential tolerance at 42 [33], 8 [34], and −1 °C [35] conditions; the transcriptome response of the inbred line B73 after 42 °C [36]; and small RNA profiling of the inbred line B73 after 38 °C stress [37]. However, few studies were systematically conducted to investigate the expression profiling under cold and heat stresses. In the present study, seedlings of maize variety B73 with a reference genome were subjected to normal conditions (25 °C, normal temperature, NT as control), cold (4 °C, extreme low-temperature, ELT; 10 °C, medium low-temperature, MLT; 16 °C, low-temperature, LT), and heat (37 °C, high-temperature, HT; 42 °C, medium high-temperature, MHT; and 48 °C, extreme high-temperature (EHT)) stress. 

We further analyzed the whole genome gene expression using the RNA-Seq technique [38]. The differentially expressed genes (DEGs) were identified through comparing the NT with the other TS samples, and these DEGs were applied to analyze the unique and common genes and the pathways responding to specific TS were identified. The weight gene co-expression network analysis (WGCNA) was conducted to investigate the specific hub genes and regulation network under cold and heat stresses. Our study advances the understanding of the molecular responses to TS in maize, which will lead to improved strategies for the development of the cold- and heat-tolerant maize varieties. 

## 2. Materials and Methods 

### 2.1. Plant Materials and Stress Treatment

We used the maize inbred line B73 with a reference genome in this study. Seeds of B73 were planted in a growth chamber with a controlled temperature (25/22 °C day/light cycle) and humidity (60% average). The growth substrate was identical to previously described methods [39]. Twelve seeds were planted in a plastic pot and eight uniform seedlings were retained for further treatments. When the seedlings were at the three leaf stage, the stress of gradient temperature included an extreme low-temperature (4 °C for 2 h, ELT), medium low-temperature (10 °C for 2 h, MLT), low-temperature (16 °C for 2 h, LT), high-temperature (37 °C for 2 h, HT), medium high-temperature (42 °C for 2 h, MHT), and extreme high-temperature (48 °C for 1 h, EHT) were applied, and seedlings grown under a normal temperature (25 °C, NT) were set as a control. To prepare the samples for sequencing, the second leaf of each seedling in each plot was collected and pooled as one replication, and three replications were prepared for each temperature point. To avoid variations in gene expression among the samples affected by circadian rhythms, TS were initiated at different times of the day, and all samples were collected in the afternoon (2:00 p.m.). In total, 21 samples were quickly frozen in liquid nitrogen and stored at −80 °C for total RNA isolation.

### 2.2. RNA Extraction, Library Preparation and RNA Sequencing

The total RNA was extracted using the TRIZOL reagent (Invitrogen, Gaithersburg, MD, USA) and was then treated with RNase-free DNaseI (Takara, Kusatsu, Japan) to remove the genomic DNA, in which DNaseI was inactivated in the presence of EDTA for incubation at 65 °C according to manufacturer’s protocol. The RNA integrity was assessed using the RNA Nano 6000 Assay Kit of the Bioanalyzer 2100 system (Agilent Technologies, Santa Clara, CA, USA). The RNA purity was checked using the NanoPhotometer spectrophotometer (IMPLEN, Westlake Village, CA, USA) and the RNA concentration was measured using the Qubit RNA Assay Kit in Qubit2.0 Fluorometer (Life Technologies, Carlsbad, CA, USA). High-quality RNA was applied to construct libraries according to the standard protocols recommended by the manufacturer. The final cDNA libraries were sequenced using the Illumina (San Diego, CA, USA) HisSeq 4000 system (paired-end 150-bp reads) by Novogene (Beijing, China). 

### 2.3. Data Filtering and Assessment

We applied the FastQC (V0.11.3) program to assess the quality of raw reads in the fastq format, and clean reads were obtained from raw reads by removing the reads containing adapters, poly-N, and low quality reads using Trimmomatic (V0.32) [40] with the default parameters. 

### 2.4. Gene Quantification and DEGs Identification

We mapped the high-quality reads onto the B73 reference genome (V4) downloaded from MaizeGDB (www.maizegdb.org) using Hisat2 (V2.0.5) [41] with the default parameters. The HTSeq tool [42] was used to count the number of fragments mapped to each gene with the parameters: -m union, -s no, and we calculated the fragments per kilobase of genes per million fragments (FPKM) for each unigene as the expression level. Differential expression analysis was performed using the DESeq2 R package to identify differentially expressed genes (DEGs) in general for transcriptomes containing biological replicates [43], and the adjusted *p*-values were calculated by the Benjamini and Hochberg method to control the false discovery rate [44]. The differential expression profiles between temperature stress conditions (ELT, MLT, LT, HT, MHT, and EHT) and the control (NT) were analyzed. Venn diagrams were drawn through the TBtools software [45]. KEGG enrichment was conducted through R package clusterProfiler [46] and volcano plots and heat maps were drawn using R packages of ggplot2 [47] and pheatmap [48], respectively. 

### 2.5. Expression Network Construction

The weighted gene co-expression network analysis (WGCNA) method [49] was applied to investigate the co-expression profiling of genes involved in TS responses and all the expressed genes were used to construct an expression matrix. Genes with similar expression patterns were clustered into the same module. The relationships between the transcripts in the module and the samples were investigated, and the important modules that were significantly associated with the samples (ELT, MLT, LT, NT, HT, MHT, and EHT) were identified. Finally, visualization of the co-expression network was performed using Cytoscape (v3.5.0) [50].

### 2.6. Analysis of Quantitative Real-Time PCR (qRT-PCR)

To validate the repeatability of the RNA-Seq data, eight DEGs were randomly selected for verification by qRT-PCR. The operation procedure was similar to as previously described by Yu et al. [51]. Briefly, the RNA samples subjected to RNA-Seq were also used for qRT-PCR, and the total RNA was purified with RNase-free DNase (Invitrogen, Gaithersburg, MD, USA) (Figure S1) following the synthesizing of single-stranded cDNA using recombinant M-MLV reverse transcriptase (Invitrogen) according to the manufacturer’s protocol. The gene-specific primers (Table S1) were designed and the qRT-PCR reaction was conducted using 2× iTaq^TM^ Universal SYBR Green Supermix (BioRad, Hercules, CA, USA). The internal reference *ZmActin1* was utilized to normalize the expression data. Relative expression levels were calculated according to the 2^-ΔΔCT^ (cycle threshold) method [52]. 

## 3. Results

### 3.1. TS Inducing Large Amount Alterations in Transcriptome

**Figure 1:**
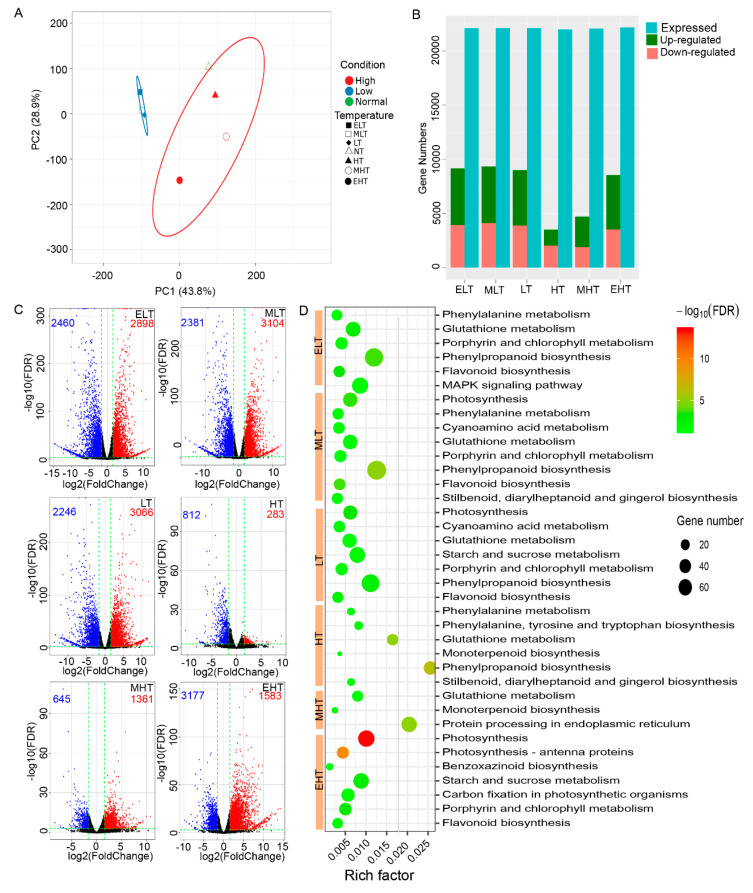
The expression characterizations of genes in response to temperature stress (TS) in maize seedling stage. (**A**) Principal component analysis (PCA) indicating the expression correlation among different temperature conditions. (**B**) The number of expressed genes and differentially expressed genes (DEGs) (*p* < 0.05, |log2(Foldchange)| > 1) under cold and heat stress. (**C**) Volcano plot indicating the DEGs with stricter standards (*p* < 0.001, |log2(Foldchange)| > 1.58). (**D**) KEGG enrichment analysis for the DEGs identified by strict standard. ELT, extreme low temperature, 4 °C; MLT, medium low temperature, 10 °C; LT, low temperature, 16 °C; NT, normal temperature, 25 °C; HT, high temperature, 37 °C; MHT, medium high temperature, 42 °C; and EHT, extreme high temperature, 48 °C.

Approximately 150 GB of clean data were totally generated from all 21 samples with the average Q30 value at approximately 96% (Table S2), which have been deposited into SRA with the accession number of PRJNA645274. These reads were mapped to the maize reference genome (Ref_Gen4) download from MaizeGDB (www.maizegdb.org) with an average mapping rate of 90%. The normalized transcription level as transcripts per kilobase per million (FPKM) was calculated, and a FPKM value of at least one sample >1 were considered as expression. A total of 22,317 expressed genes were detected in all of these samples (Table S3), and the average FPKM was calculated for ELT, MLT, LT, NT, HT, MHT, and EHT. Principal component analysis (PCA) showed that the gene expressions under ELT, MLT, and LT were well clustered and separated with NT and heat-stress whereas the gene expressions under HT, MHT, and EHT exhibited larger variations (Figure 1A), implying a differential response to cold and heat stress. 

We first identified the DEGs of the stress conditions from NT based on the following criteria: (1) |log2(foldchange)| > 1, (2) *p* < 0.05. A large amount of DEGs were discovered in ELT (9166), MLT (9235), LT (9027), and EHT (8559), most of which were up-regulated (Figure 1B). To obtain high-confidence DEGs involved in temperature stress, more strict standards, including |log2(foldchange)| > 1.58 and *p* < 0.001 were used. The number of DEGs finally ranged from 1097 in HT to 5485 in MLT and the number of DEGs under cold stress (ELT, MLT, and LT) were comparative with MHT, whereas HT and MHT had fewer DEGs (Figure 1C, Supplemental Data Set S1). KEGG enrichment analysis demonstrated that 16 different metabolic pathways were significantly enriched in ELT, MLE, LT, HT, MHT, and EHT (Figure 1D). The most enriched pathways were the glutathione metabolism, phenylpropanoid biosynthesis, flavonoid biosynthesis, porphyrin and chlorophyll metabolism, and photosynthesis, indicating that these were the major pathways responding to TS. 

### 3.2. Common and Specific DEGs Responded to TS

**Figure 2:**
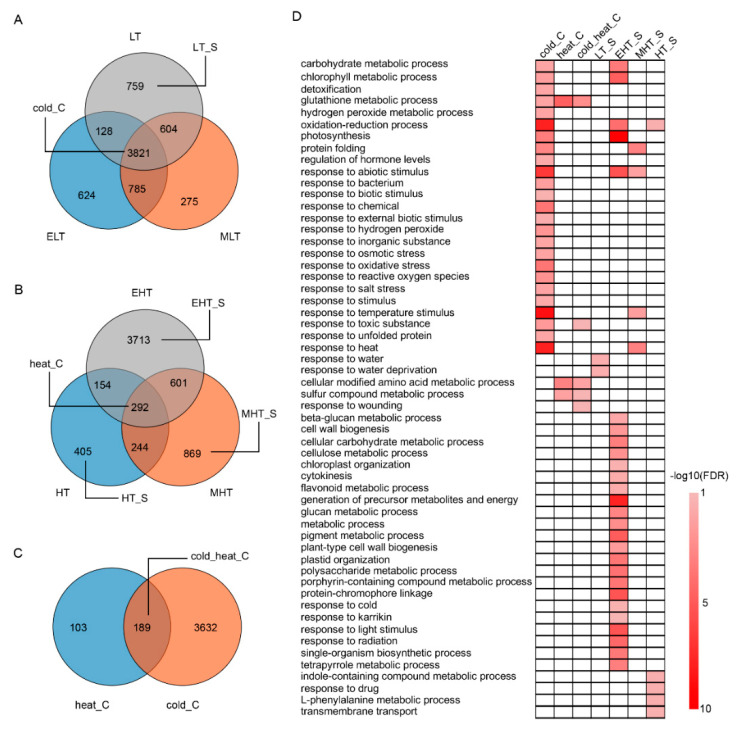
Comparison of differentially expressed genes (DEGs) and the corresponding gene ontology (GO) terms in response to cold and heat stress in maize seedlings. (**A**) Comparison of DEGs in response to cold stress. (**B**) Comparison of DEGs in response to heat stress. (**C**) Comparison of DEGs in response to cold and heat stress. (**D**) GO terms of the common and specific DEGs in response to cold and heat stress. C, common; S, specific. ELT, extreme low temperature, 4 °C; MLT, medium low temperature, 10 °C; LT, low temperature, 16 °C; NT, normal temperature, 25 °C; HT, high temperature, 37 °C; MHT, medium high temperature, 42 °C; and EHT, extreme high temperature, 48 °C.

For DEGs under cold stress, over 54% of DEGs (3821 of 6996) were common (C) for cold (cold_C), and only 759, 275, and 624 DEGs were specific (S) for LT (LT_S), MLT (MLT_S), and ELT (ELT_S), respectively (Figure 2A). However, less than 5% DEGs (292 of 6278) were common for heat (heat_C) and approximately 80% DEGs were specific for heat stress, in which 3713, 869, and 405 were specific for EHT (EHT_S), MHT (MHT_S), and HT (HT_S), respectively (Figure 2B). A total of 189 DEGs were common for cold and heat stress (cold_C vs. heat_C) (Figure 2C), which included two AP2/EREBP family genes (*Zm00001d023535* and *Zm00001d027925*), two auxin responsive proteins (*Zm00001d033462* and *Zm00001d041418*), two gibberellin oxidase genes (*Zm00001d032223* and *Zm00001d04341*1), one heat stress transcription factor (*Zm00001d034433*), seven genes (*Zm00001d029696*, *Zm00001d024839*, *Zm00001d018220*, *Zm00001d048558*, *Zm00001d018809*, *Zm00001d042104*, and *Zm00001d043344*) encoding glutathione transferase, and five peroxidase genes (*Zm00001d002898*, *Zm00001d042022*, *Zm00001d053554*, *Zm00001d034129*, and *Zm00001d022457*). Gene ontology (GO) analysis demonstrated that differential functional classifications represented cold and heat stress responses (Figure 2D). The DEGs of cold_C were mainly enriched in the oxidation-reduction process, photosynthesis, protein folding, and the response to abiotic stress, particularly for temperature stimulus, and the DEGs of heat_C and cold_heat_C were enriched in the hydrogen peroxidase metabolic process, cellular modified amino acid metabolic process, and sulfur compound metabolic process. For the stress specific DEGs, the significant enrichment of GO terms were discovered in EL_S, EHT_S, MHT_S, and HT_S. Twenty-seven GO terms were enriched for DEGs of EHT_S, including photosynthesis, the response to abiotic stimulus, the chlorophyll metabolic process, the generation of precursor metabolites and energy, the pigment metabolic process, protein-chromophore linkage, and the oxidation-reduction process, whereas fewer GO terms were enriched in EL_S, EHT_S, and HT_S.

### 3.3. Ca^2+^ and ROS Signaling Were Induced under TS

**Figure 3:**
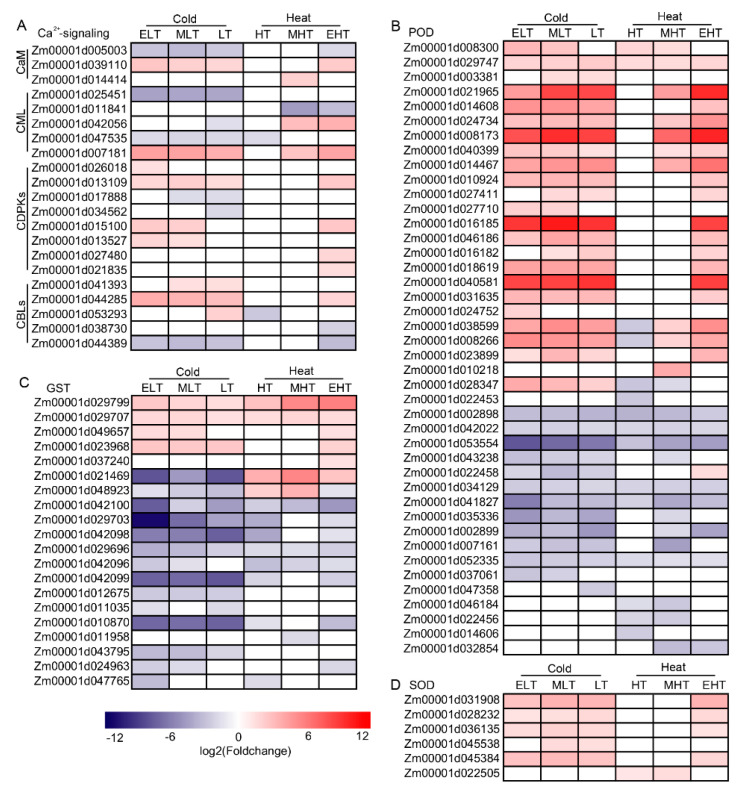
Heatmap of the differentially expressed genes (DEGs) involved in Ca^2+^ signaling and ROS-scavenging in response to temperature stress. (**A**) The heatmap of DEGs involved in Ca^2+^ signaling. (**B**) The heatmap of DEGs encoded GST. (**C**) The heatmap of DEGs encoded POD. (**D**) The heatmap of DEGs encoded SOD. CaM, calmodulin, CML, CaM-like proteins; CDPKs, Ca^2+^-dependent protein kinases; CBLs, calcineurin B-like proteins; POD, peroxidase; GST, glutathione S-transferase; superoxide dismutase. ELT, extreme low temperature, 4 °C; MLT, medium low temperature, 10 °C; LT, low temperature, 16 °C; NT, normal temperature, 25 °C; HT, high temperature, 37 °C; MHT, medium high temperature, 42 °C; and EHT, extreme high temperature, 48 °C.

The investigations in rice and *Arabidopsis* demonstrated that Ca^2+^ signaling and ROS play critical roles in signal perception and transduction [11,12]. We analyzed the DEGs involved in Ca^2+^ signaling, including calmodulin (CaM), CaM-like proteins (CML), Ca^2+^-dependent protein kinases (CDPKs), and calcineurin B-like proteins (CBLs) (Figure 3A). A total of 21 DEGs were related Ca^2+^ signaling, the majority of which were up-regulated after cold- or heat-treatment. One CaM (*Zm00001d039110*), one CML (*Zm00001d007181*), two CDPKs (*Zm00001d013109* and *Zm00001d015100*), and one CBL (*Zm00001d044285*) were up-regulated under cold and heat stress, indicating that these genes may be involved in the two stresses. The genes encoding enzymes involved in reactive oxygen species (ROS) metabolism were also differentially expressed, mainly including peroxidase (POD), glutathione S-transferase (GST), and superoxide dismutase (SOD). Forty-two PODs were differentially expressed, and over half of them were up-regulated under cold and heat conditions, whereas a small number of PODs were specifically expressed in cold or heat stress (Figure 3B). Twenty GSTs were induced and the majority of them were down-regulated (Figure 3C). Four genes (*Zm00001d029799*, *Zm00001d029707*, *Zm00001d049657*, and *Zm00001d023968*) were up-regulated while Zm00001d021469 displayed the opposite expressions under cold and heat conditions. Six genes encoding SOD (*Zm00001d031908*, *Zm00001d028232*, *Zm00001d036135*, *Zm00001d045538*, *Zm00001d045384*, and *Zm00001d022505*) were up-regulated in both conditions but the majority of them were only induced by EHT (Figure 3D).

### 3.4. Dynamic Expression of Transcription Factors in Response to TS

**Figure 4:**
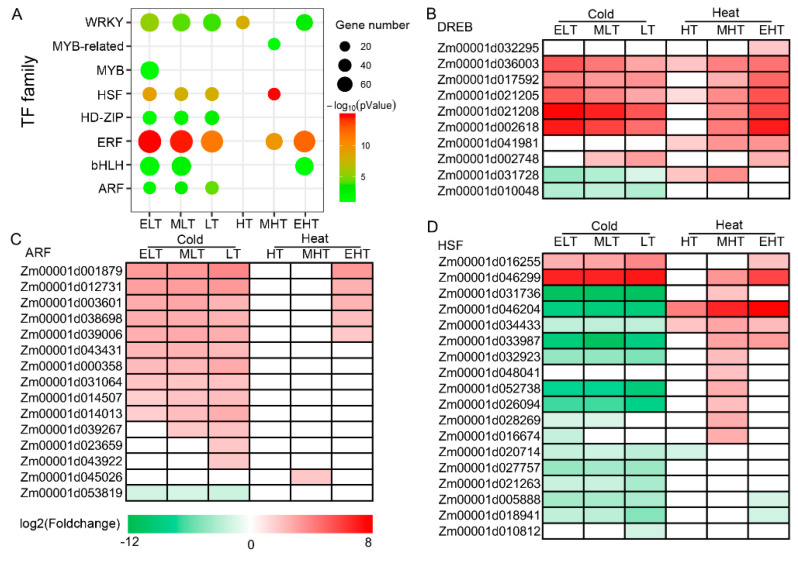
The enrichment analysis of transcription factors (TFs) after temperature stress (TS). (**A**) The bubble chart shows the significantly enriched TF families in response to TS. (**B**) The gene list of DREB TFs enriched after TS treatment. (**C**) The gene list of ARF TFs enriched after TS treatment. (**D**) The gene list of HSF TFs enriched after TS treatment. ELT, extreme low temperature, 4 °C; MLT, medium low temperature, 10 °C; LT, low temperature, 16 °C; NT, normal temperature, 25 °C; HT, high temperature, 37 °C; MHT, medium high temperature, 42 °C; and EHT, extreme high temperature, 48 °C.

Transcription factors play vital roles in regulating gene expression in responding to stress conditions, such as heat stress transcription factors, including *HsfA1* [27,28,29]. Enrichment analysis of transcription factor (TF) families demonstrated that eight TF families were significantly enriched in at least one stress condition (Figure 4A). Under cold conditions, ARF, ERF, HD-ZIP, HSF, and WRKY were significantly enriched under ELT, MLT, and LT, whereas bHLH was enriched under ELT and MLT, and MYB was enriched under ELT. The relative TF families were enriched under heat conditions, which included ERF under MHT and EHT, bHLH and WRKY under EHT, HSF and MYB-related under MHT, and WRKY under HT, indicating that different TFs may be involved in TS with differential degrees. We further investigated the differentially expressed TFs (Figure 4B–D, Figures S1–S8). 

Drought-responsive element proteins (DREBs), belonging to the ERF family, are the important regulators involved in cold and heat stresses [11,12]. Ten DREBs were differentially expressed, 9 of 10 were up-regulated under heat conditions, and six (*Zm00001d036003*, *Zm00001d017592*, *Zm00001d021205*, *Zm00001d021208*, *Zm00001d002748*, and *Zm00001d002618*) were up-regulated under cold and heat conditions (Figure 4B). Except for DREBs, 75 ERFs were differentially expressed after cold and heat stress, of which many genes, such as *Zm00001d051451*, *Zm00001d008872*, *Zm00001d018191*, and *Zm00001d039424*, were up-regulated in both conditions (Figure S2). A total of 15 ARFs were identified, and 14 of 15 were up-regulated, most of which were induced under cold conditions (Figure 4C). The majority of HSFs were up-regulated under heat conditions and down-regulated under cold conditions whereas two HSFs (*Zm00001d016255* and *Zm00001d046299*) were up-regulated under both conditions (Figure 4D). Most of the TFs in these enriched families, such as bHLH (Figure S3), HD-ZIP (Figure S4), MYB (Figure S5), and MYB-related (Figure S6), were also up-regulated after TS but over half of the WRKYs (Figure S7) were down-regulated. Furthermore, the majority of the differentially expressed bZIP (Figure S8) and NAC (Figure S9) were also up-regulated. These results collectively demonstrated that induced expression of TFs participated in cold and heat responses. 

### 3.5. Hormone Metabolism Was Significantly Induced

**Figure 5:**
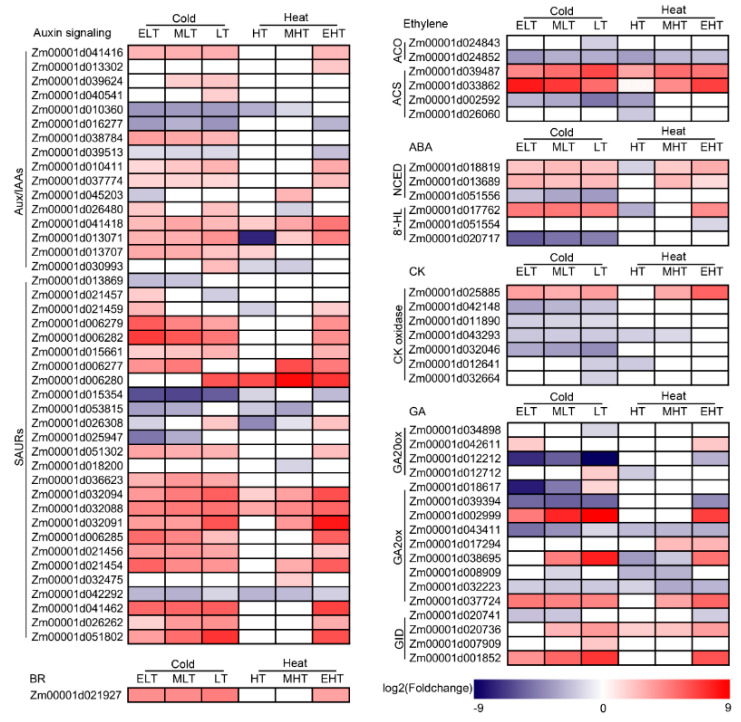
Heatmap of the differentially expressed genes involved in hormone signaling in response to temperature stress. ACS, 1-aminocyclopropane-1-carboxylate (ACC) synthesis (ACS); ACO, ACC oxidase; NCED, 9-cis-epoxycarotenoid dioxygenase; GA2ox, GA2 oxidase; GA20ox, GA20 oxidase; GID, gibberellin insensitive dwarf; and BR, brassinosteroids. ELT, extreme low temperature, 4 °C; MLT, medium low temperature, 10 °C; LT, low temperature, 16 °C; NT, normal temperature, 25 °C; HT, high temperature, 37 °C; MHT, medium high temperature, 42 °C; and EHT, extreme high temperature, 48 °C.

Hormone biosynthesis, metabolism, and signaling were seriously affected by stress conditions and, in turn, regulated the plant tolerances to stresses. The DEGs of auxin signaling, ethylene, abscisic acid (ABA), cytokinins (CK), gibberellins (GA), and brassinosteroids (BR) biosynthesis and metabolism were identified (Figure 5). Auxin could rapidly induce three family genes, including auxin/indole-2-acetic acid inducible (Aux/IAAs), Grethchen Hagen 3 (GH3), and SAURs, in which 16 Aux/IAAs and 26 SAURs were differentially expressed. Twenty of these DEGs, such as *Zm00001d041418* and *Zm00001d032094*, were up-regulated whereas only six DEGs were down-regulated under both cold and heat conditions. Two key enzymes, 1-aminocyclopropane-1-carboxylate (ACC) synthesis (ACS) and ACC oxidase (ACO), were involved in ethylene biosynthesis [53], and two ACSs (*Zm00001d039487* and *Zm00001d033862*) were up-regulated under cold and heat conditions. Three genes encoding 9-cis-epoxycarotenoid dioxygenase (NCEDs), that is, the first rate-limiting enzyme in ABA bio synthesis [54], were differentially expressed, and two (*Zm00001d018819* and *Zm00001d013689*) were up-regulated under cold and heat conditions except in HT. Three genes encoding enzymes of ABA catabolism (8′-hydroxylase, 8′-HL) [55] were detected and only *Zm00001d025885* was up-regulated in ELT, MLT, LT, and EHT. Seven genes encoding CK oxidase that irreversibly degraded cytokinins with an unsaturated side chain were differentially expressed, which mainly responded to cold conditions. Thirteen genes involved in GA biosynthesis, including GA20 oxidase (GA20ox) and GA2 oxidase (GA2ox), were significantly induced, in which *Zm00001d002999* and *Zm00001d037724* were up-regulated under cold and heat conditions, and three (*Zm00001d020736*, *Zm00001d007909*, and *Zm00001d001852*) of four GA receptors, gibberellin insensitive dwarfs (GIDs), were also up-regulated. One gene encoding BES1/BZR1 protein (*Zm00001d021927*) involved BR signaling was also significantly up-regulated under cold and EHT conditions. These results indicated that the hormone metabolism was significantly induced under cold and heat conditions.

### 3.6. Heat Shock Proteins (HSPs) Were Mainly Up-Regulated under Heat Conditions

**Figure 6:**
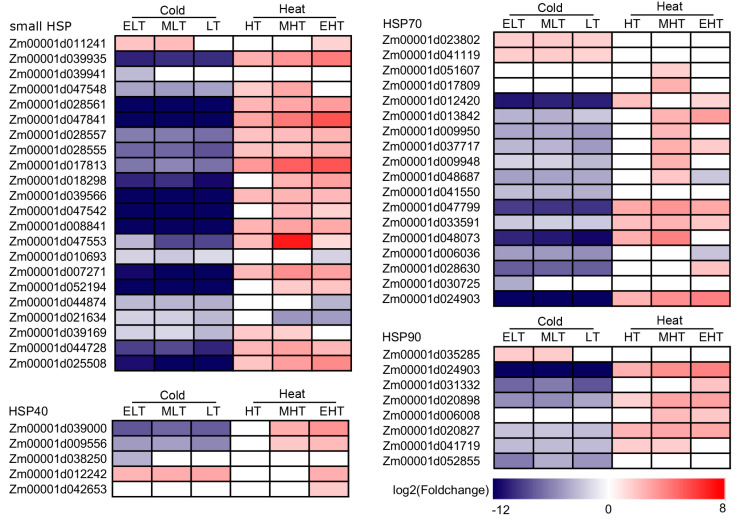
Heatmap of the differentially expressed genes encoding the members of HSP family in response to temperature stress. ELT, extreme low temperature, 4 °C; MLT, medium low temperature, 10 °C; LT, low temperature, 16 °C; NT, normal temperature, 25 °C; HT, high temperature, 37 °C; MHT, medium high temperature, 42 °C; and EHT, extreme high temperature, 48 °C.

HSPs are major functional proteins for cellular homeostasis, protein conformation, folding, and stabilization under stress conditions [56,57,58,59]. A total of 22, 5, 18, and 8 of small HSP, HSP40, HSP70, and HSP90 were differentially expressed, respectively, in which almost all of these HSPs were up-regulated under heat conditions and most of these HSPs were down-regulated under cold conditions (Figure 6). Only one small HSP (*Zm00001d011241*), one HSP40 (*Zm00001d012242*), two HSP70 (*Zm00001d023802* and *Zm00001d041119*), and one HSP90 (*Zm00001d035285*) were up-regulated under cold conditions, whereas only two HSP20 and one HSP70 were down-regulated under cold and heat conditions, indicating that HSPs were mainly up-regulated under heat conditions in maize. 

### 3.7. The Hub Genes Associated with Cold and Heat Stress

**Figure 7:**
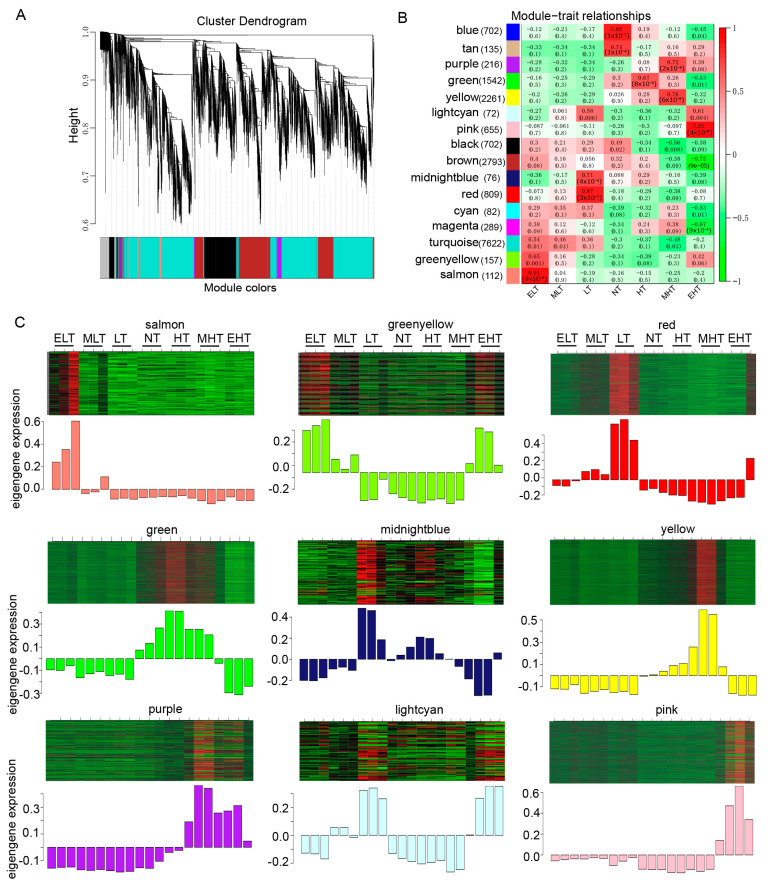
Co-expression network analysis of transcripts involved in temperature stress (TS) in maize seedling leaves. (**A**) The weighted gene co-expression network (WGCNA) identified 16 modules presented by hierarchical cluster tree and color bands. (**B**) Analysis of the module–trait association. Each row represents a module, and each column represents a sample under TS conditions. The numbers on the top and bottom of each cell represent the correlation and significant p-values, respectively. The number of genes in each module is presented in the bracket. (**C**) Heatmap indicating the eigengene expression profile for nine significantly associated modules in ELT, MLT, LT, NT, HT, MHT, and EHT. ELT, extreme low temperature, 4 °C; MLT, medium low temperature, 10 °C; LT, low temperature, 16 °C; NT, normal temperature, 25 °C; HT, high temperature, 37 °C; MHT, medium high temperature, 42 °C; and EHT, extreme high temperature, 48 °C.

To investigate the hub genes involved in cold and heat stress, WGCNA was performed and we obtained 16 distinct modules as shown in the dendrogram, in which the major tree branches are labeled with different colors to determine the modules (Figure 7A). The associations between the modules and distinct samples were calculated and the gene numbers ranged from 72 to 7622, with 9 modules significantly positively (*p* < 0.05, *r* > 0.6) correlated with cold- or heat-stress (Figure 7B). For example, the ‘salmon’ module with 112 genes was significantly associated with ELT (*p* = 9 × 10^−9^, *r* = 0.91), the ‘red’ module with 809 genes was significantly associated with LT (*p =* 3 × 10^−7^, *r* = 0.87), and the ‘pink’ module with 655 genes was significantly associated with EHT (*p* = 4 × 10^−9^, *r* = 0.92). These modules had specific eigengene expressions, for instance ‘salmon’ was specifically up-regulated under ELT, and ‘red’ was specifically up-regulated under LT (Figure 7C).

**Figure 8:**
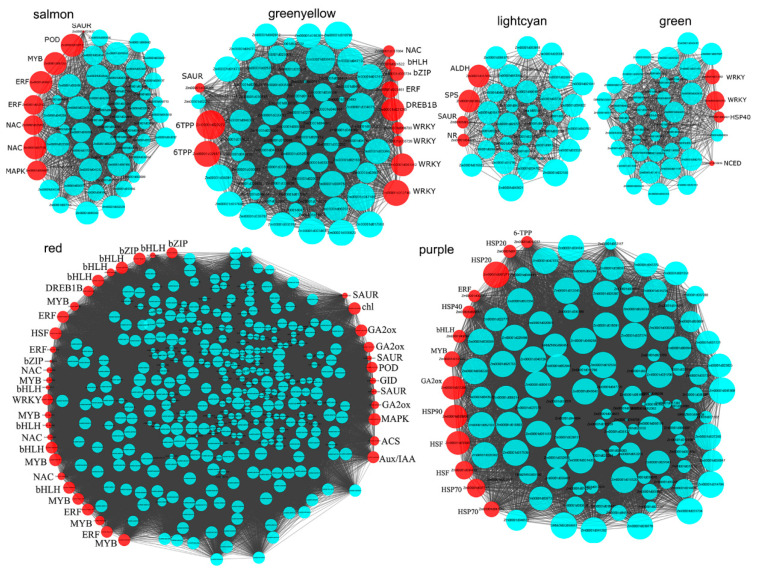
The correlation network of DEGs in six significantly associated modules (salmon, green-yellow, light-cyan, green, red, and purple). The size of circles relates to the higher connectivity degree in each module. The red circles represent the putative important candidate genes. ERF, NAC, bHLH, bZIP, DREB1B, WRKY, MYB, and HSF were different transcription factor families, and HSP20, HSP40, HSP70, and HSP90 belong to the HSP family. POD, peroxidase; 6TPP, trehalose-6-phosphate phosphatase; SPS, sucrose-phosphate synthase; NR, nitrate reductase; NCED, 9-cis-epoxycarotenoid dioxygenase; chl, chlorophyllase; GA2ox, GA2 oxidase; GID, gibberellin insensitive dwarf; and ACS, 1-aminocyclopropane-1-carboxylate synthesis.

The DEGs and their connectivity in these nine modules were the most interesting (Table S4), indicating that the hub genes were involved in cold- and heat-stress. In the ‘greenyellow’ module, a total of 79 genes were differentially expressed under ELT, and *Zm00001d0200272*, encoding trehalose-6-phosphate phosphatase (6TPP) involved in the starch and sucrose metabolism, had the highest degree of connectivity (77) (Figure 8, Table S4). The degree of connectivity of *Zm00001d021205*, encoding the dehydration-responsive element-binding protein 1B also called C-repeat binding factor 2 (CBF2), was relatively higher (69). The transcription factors, such as NAC (*Zm00001d017084*), bHLH (*Zm00001d024522*), bZIP (*Zm00001d008734*), ERF (*Zm00001d022461*), and four WRKYs were also co-expressed. The ‘salmon’ module was also associated with ELT, the hub genes mainly encoded TFs, such as NACs, ERFs, MYB, POD, and genes involved in MAPK signaling (Figure 8, Table S4). 

The ‘red’ module was significantly associated with LT, and a large amount of TFs, including HSF (*Zm00001d016255*), DRED1B (*Zm00001d002748*), ERF, bHLH, NAC, bZIP, and MYB (*Zm00001d032926*), were involved in the chlorophyll metabolism, and genes involved in hormone signaling and metabolism, such as GA2ox, SUAR, and ACS, were co-expressed to respond LT (Figure 8, Table S4). The ‘green’ module was significantly associated with HT, in which two WRKYs (*Zm00001d032265* and *Zm00001d020955*), one HSP40 (*Zm00001d002501*), and one NCED (*Zm00001d018819*) were significantly up-regulated (Figure 8, Table S4). 

Two modules, ‘yellow’ and ‘purple’, were significantly associated with MHT, and they contained 786 and 118 DEGs, respectively (Figure 8, Table S4). In the ‘purple’ module, HSP family proteins, such as HSP20 (*Zm00001d017813* and *Zm00001d007271*), HSP40 (*Zm00001d009556*), HSP70 (*Zm00001d037717* and *Zm00001d047452*), and HSP90 (*Zm00001d020898*); TFs, including HSFs (*Zm00001d033987* and *Zm00001d034433*), MYB, ERF, and bHLH; and the gene encoding 6TPP, were up-regulated. In ‘yellow’, three glutathione transferase genes (*Zm00001d048923*, *Zm00001d021469*, and *Zm00001d048559*), one DREB1B (*Zm00001d031728*), five HSP70 (*Zm00001d048073*, *Zm00001d017809*, *Zm00001d009950*, *Zm00001d009948*, and *Zm00001d051607*), and four HSFs (*Zm00001d052738*, *Zm00001d032923*, *Zm00001d048041*, and *Zm00001d026094*) had over 750 connectivities (Table S4). Furthermore, ‘pink’ and ‘lightcyan’ were significantly correlated with EHT, and 461 genes in ‘pink’ were DEGs, in which the genes with highest degree of connectivity were mainly involved in photosynthesis, including *Zm00001d026599*, *Zm00001d048998*, *Zm00001d001857*, *Zm00001d039040*, *Zm00001d044402*, *Zm00001d050403*, *Zm00001d007267*, and *Zm00001d013146* (Table S4). However, the genes encoding sucrose-phosphate synthase (SPS), aldehyde dehydrogenase (ALDH), nitrate reductase (NR), and SAUR in the ‘lightcyan’ module responded to EHT (Figure 8, Table S4). These candidates are the possible hub genes involved in TS.

### 3.8. Validation of RNA-Seq Analysis by qRT-PCR

**Figure 9:**
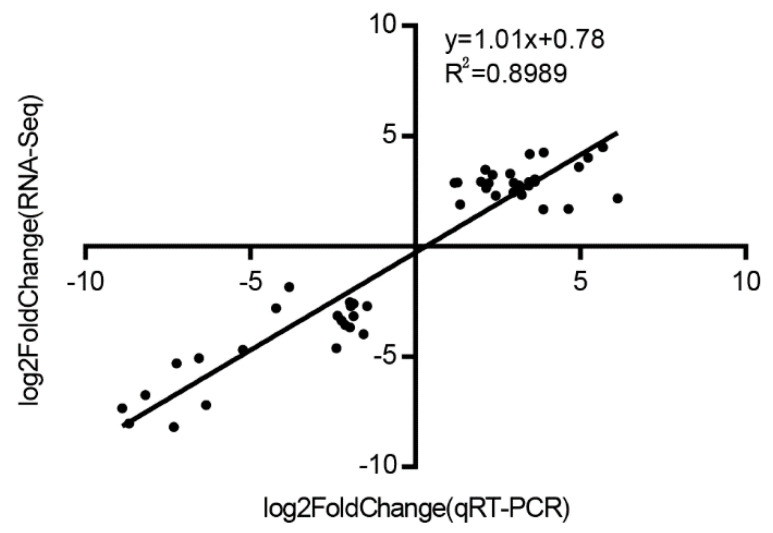
The expression correlation of eight DEGs between quantitative real-time PCR (qRT-PCR) and RNA-Seq data under cold and heat stresses.

To verify the reliability of the RNA-Seq data in maize seedlings, the expression of eight genes under ELT, MLT, LT, NT, HT, MHT, and EHT were analyzed using qRT-PCR. The ratio of the expression levels between NT and TS was calculated and compared with the foldchange obtained from RNA-Seq. A high significant correlation (*R*^2^ = 0.8989, *n* = 48) between RNA-Seq and qRT-PCR data was observed (Figure 9), which confirmed the authenticity of the DEGs in this study.

## 4. Discussion

The frequency of extreme temperatures, such as low and high temperatures is increasing worldwide due to climate change, which are becoming the major limitations for maize growth, development, and yield. The tropically-originating maize diffused to temperature climates [1] and easily experienced cold stress conditions, for example, maize seedlings grown in Northeast China often encountered a cold spell in late spring. Maize is also sensitive to heat stresses that were encountered by seedlings grown in the summer corn area and pollination and silking in the spring corn area in China. The degree of TS largely fluctuated at different planting regions and in different growth years. Therefore, it is important to prevent TS damage through maize cultivars with superior tolerance followed by understanding the complex molecular mechanisms of maize responses to TS. 

In the present study, the B73 seedlings were exposed to gradients of cold- and heat-stress to investigate the potential regulation network underlying TS in maize, and a large amount of DEGs with responses to TS were discovered (Figure 1). The DEGs under cold conditions (ELT, 5358; MLT, 5485; and LT, 5312) had a significantly higher number than under heat conditions (HT, 1095; and MHT, 2006) while the DEGs under EHT (4760) had relatively high amount, indicating that maize seeding is highly sensitive to cold stress and extreme high temperature. Although the HT (37 °C) condition is common in the maize life cycle, DEGs were also identified to respond to it. The PCA and DEGs analysis also showed the large variations of gene expression under heat stress (Figure 1 and Figure 2), implying more complex regulation of different degrees of heat stress. KEGG and GO analysis demonstrated the common and divergent metabolism pathways involved in cold and heat stress.

Cold and heat stresses were at lower and higher temperatures than the growth thresholds, respectively. As the TS, similar negative effects of cold and heat stresses occurred, which included the inhibition of seed germination, restrictions in the plant growth, affecting the reproduction, and reducing the yield [60]. The similar adverse effects of cold and heat stresses were reflected in the biochemical and molecular impacts, such as transducing the TS signal, changing the fluidity of cell membranes [61], and affecting the activities of ROS-scavenging enzymes [62,63,64]. 

Common characterizations involved in TS in *Arabidopsis* include Ca^2+^ signaling and ROS, which could induce downstream actions, such as the expression of core-regulators in the cold- and heat-signaling pathways [11,12,65]. The Ca^2+^ signal is recognized by calcium-binding proteins, including CaM, CML, CDPKs, and CBLs, and 21 DEGs were discovered in the present investigation (Figure 3). The majority of these Ca^2+^ signaling-related genes were up-regulated under TS, and five DEGs, such as Zm00001d039110 encoding CaM, *Zm00001d007181* encoding CML, and *Zm00001d013109* CDPKs, were up-regulated under both cold and heat stress. ROS was induced by TS and tightly controlled in plants because excess ROS are harmful. The 68 genes encoding ROS scavenging enzymes, such as POD, GST, and SOD were differentially expressed, and most of them had a similar expression tendency (Figure 3). For example, most of genes encoding POD were up-regulated, and most of genes encoding GST were down-regulated under both cold and heat conditions. These results indicated that possible similar regulations of TS signaling and ROS at the molecular level were presented in maize seedlings.

Ca^2+^ signaling activated by cold stress triggered the expression of CBFs, which is involved in a well-studied cold regulation pathway called the CBF-dependent pathway in *Arabidopsis* [15,16]. The accumulated evidence demonstrated that hormones interact with the CBF pathway to regulate the cold response [66], for instance CBF1 reduces bioactive GA levels [67], BR-regulated BES1 activates the expression of CBFs [68], ET-regulated EIN3 represses CBF gene expression [21], and auxin plays an important role for the survival of recovery growth [69]. 

Five genes (*Zm00001d017592*, *Zm00001d021205*, *Zm00001d021208*, *Zm00001d002618*, and *Zm00001d002748*) encoding DERB1 were significantly up-regulated under cold stress (Figure 4), and 42, 5, 6, 7, and 17 DEGs were involved in auxin, ET, ABA, CK, and GA signaling (Figure 5), respectively, and a co-expression model (‘red’) of DERB1 and hormones was detected (Figure 8), implying that hormones were also participants in the cold-induced CBF-dependent pathway in maize seedling leaves. Notably, 15 ARFs, transcription factor of auxin response factors, were differentially expressed after cold stress, and 13 ARFs (such as *Zm00001d043431*, *Zm00001d014013*, *Zm00001d014507*, and *Zm00001d000358*) were up-regulated (Figure 4). In addition, 28 of 42 DEGs involved in auxin signaling were also up-regulated under cold conditions, indicating that auxin is an important hormone in response to cold stress in maize seedlings. 

HSFs belong to a conserved transcription factor family and bind to downstream genes encoding transcription factors, enzymes, and chaperone proteins [12]. HsfA1s have critical roles in response to heat stress and appear as the master regulators to activate the transcriptional networks in *Arabidopsis* [27,60]. HSFs could rapidly induce the expression of HSPs, and the small HSPs, HSP60, HSP70, HSP90, and HSP100 in *Arabidopsis* have shown functions in heat tolerance [70]. HSP70 and HSP90 interact with HsfA1s to inhibit its activity under normal conditions, whereas HsfA1s is released from HSP70/90 to be activated under heat stress [71,72]. 

A total of 18 HSFs were identified as DEGs, of which 12 DEGs, such as *Zm00001d046299*, *Zm00001d031736*, *Zm00001d046204*, and *Zm00001d034433*, were up-regulated in heat stress (Figure 4), indicating that HSFs were significantly induced by heat stress. Forty-one of 53 HSPs were up-regulated by heat stresses, which included 18, 4, 13, and 6 genes encoding small HSP, HSP40, HSP70, and HSP90, respectively (Figure 6). The co-expression network analysis also demonstrated that many genes in the modules ‘yellow’ and ‘purple’ encoding HSFs (such as *Zm00001d052738*, *Zm00001d032923*, *Zm00001d048041*, and *Zm00001d026094*) and HSPs (such as Zm00001d048073, *Zm00001d017809*, *Zm00001d009950*, *Zm00001d009948*, and *Zm00001d051607*) had similar expression characteristics, which were significantly associated with heat stress (Figure 7 and Figure 8, Table S4), indicating that regulatory networks mediated by HSFs and HSPs were also evoked in response to heat stress in maize seedlings.

Transcription factors are involved in various growth, development, and stress response processes. In addition to the core regulators of cold (DREB1) and heat (HSFs) stress, a large amount of TFs were differentially expressed under TS, including ERFs, NACs, MYB, HD-ZIP, WRKY, and bHLH (Figure 3, Figures S1–S8), which were similar to the previous investigation of cold (4 °C) and heat (42 °C) stress imposed on B73 seedlings [73], indicating that these TFs play vital roles in the TS responses. A fraction of TFs, including 5 DERBs (Figure 3B), 2 HSFs (Figure 3D), 5 ARFs (Figure 3C), 30 ERFs (Figure S1), and 24 bHLHs (Figure S2), were up-regulated under both cold and heat conditions. These TFs also presented similar expression profiling with the core regulators of TS (Figure 8), implying their possible functions involved in TS regulation. 

Our results presented here generated a relatively robust list of genes that respond to temperature stresses at the maize seeding stage, which will likely be useful for future functional genomics research and precipitate more comprehensive studies on gene regulation under cold and heat conditions. These findings contribute new knowledge to our understanding of temperature stress regulatory network and will facilitate the future engineering of temperature stress tolerant maize varieties.

## Supplementary Materials

The following are available online at https://www.mdpi.com/2073-4425/11/8/881/s1, Figure S1: The gel image of the DNaseI-degisted RNA samples. The number 1 to 21 represents ELT-1, ELT-2, ELT-3, MLT-1, MLT-2, MLT-3, LT-1, LT-2, LT-3, NT-1, NT-2, NT-3, HT-1, HT-2, HT-3, MHT-1, MHT-2, MHT-3, EHT-1, EHT-2, and EHT-3, respectively. M, marker, Figure S2: Heatmap indicating the gene list of ERF TFs enriched after temperature stress, Figure S3: Heatmap indicating the gene list of bHLH TFs enriched after temperature stress, Figure S4: Heatmap indicating the gene list of HA-ZIP TFs enriched after temperature stress, Figure S5: Heatmap indicating the gene list of MYB TFs enriched after temperature stress, Figure S6: Heatmap indicating the gene list of MYB-related TFs enriched after temperature stress, Figure S7: Heatmap indicating the gene list of WRKY TFs enriched after temperature stress, Figure S8: Heatmap indicating the gene list of bZIP TFs enriched after temperature stress, Figure S9: Heatmap indicating the gene list of NAC TFs enriched after temperature stress, Table S1: The primers of eight differentially expressed genes used for qRT-PCR verification, Table S2: Data information of transcriptome sequencing, Table S3: The FPKM values of each sample of 22,317 expressed genes, Table S4: The differentially expressed genes presented in nine different modules, Supplemental Data Set S1: The list of differentially expressed genes after temperature stresses.
