# Supplementary figures and images for: Transcriptomic Analysis Revealed the Common and Divergent Responses of Maize Seedling Leaves to Cold and Heat Stresses
Source: Genes (Basel). 2020 Aug 3;11(8):881. doi: 10.3390/genes11080881 (PMC7464670; doi:10.3390/genes11080881)

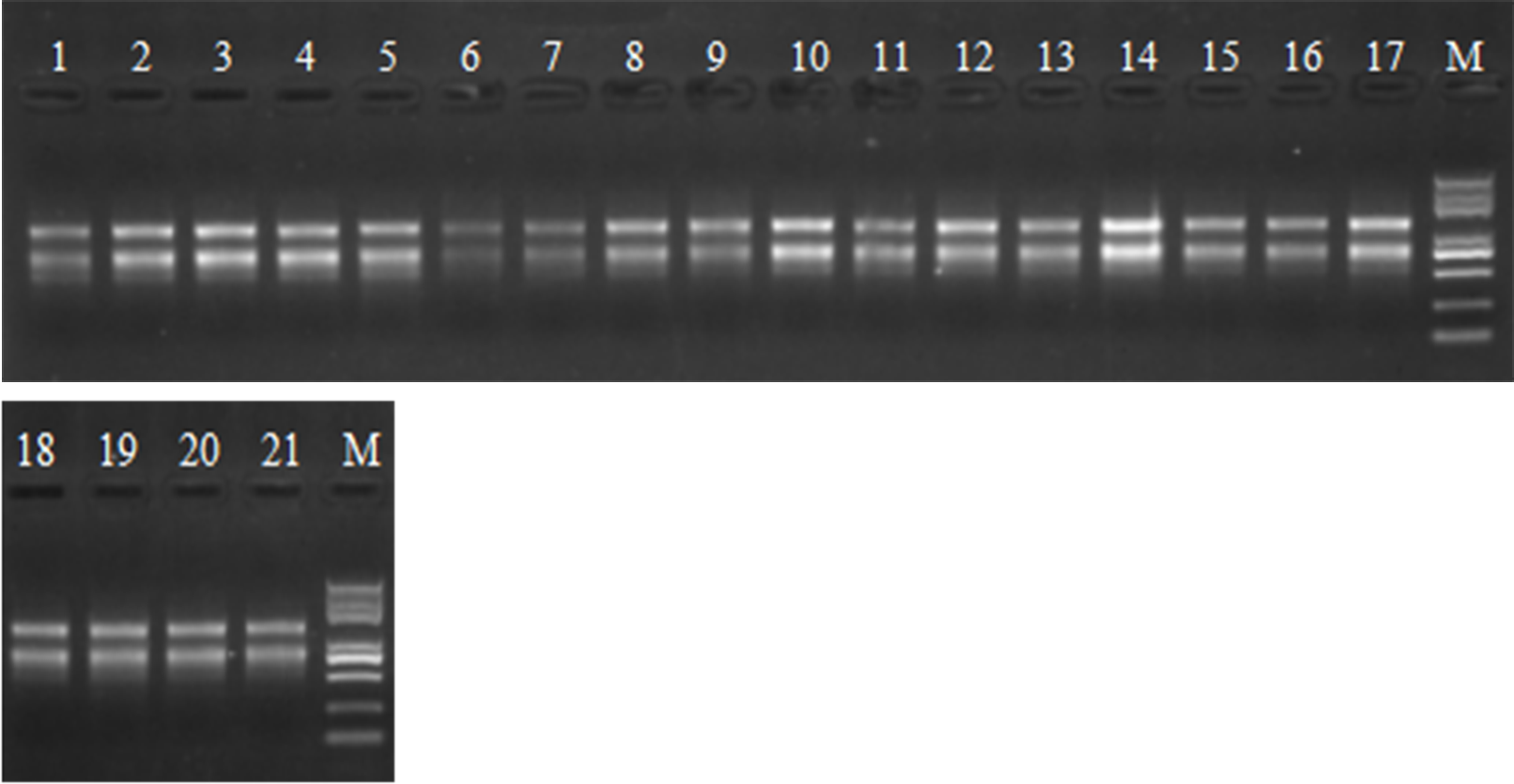

Supplement: Supplementary file 1 [file genes-11-00881-s001.zip › Supplemental Materials/Figure S1.tif]

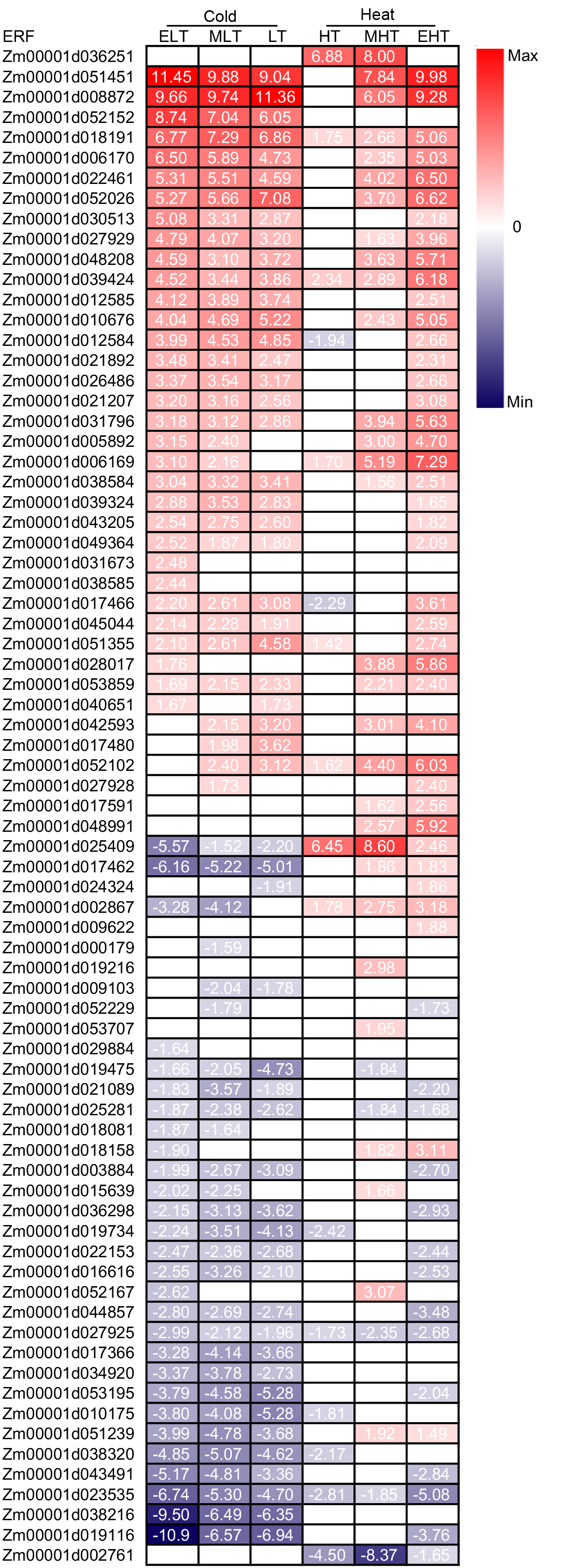

Supplement: Supplementary file 1 [file genes-11-00881-s001.zip › Supplemental Materials/Figure S2-ERF.tif]

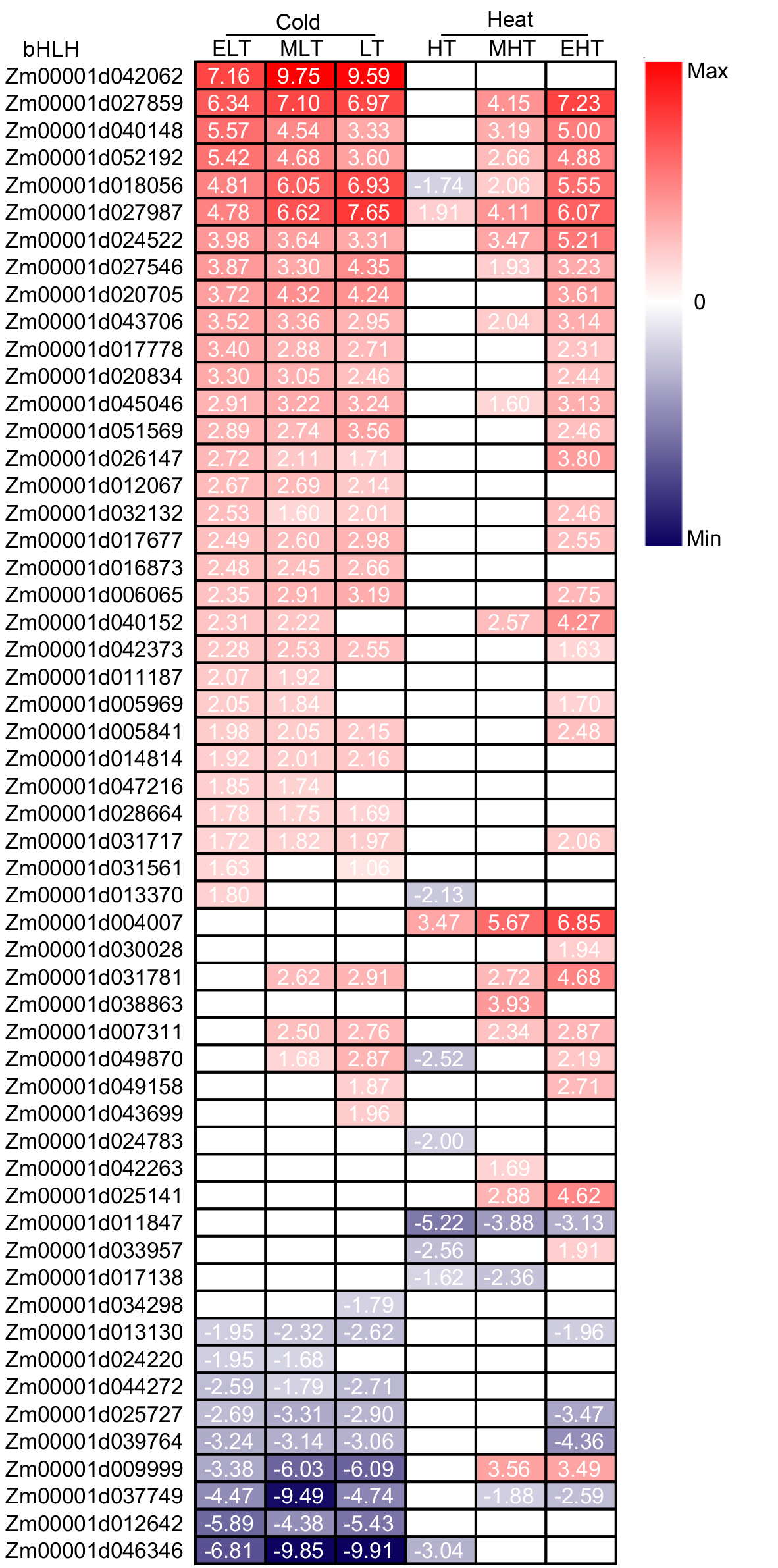

Supplement: Supplementary file 1 [file genes-11-00881-s001.zip › Supplemental Materials/Figure S3-bHLH.tif]

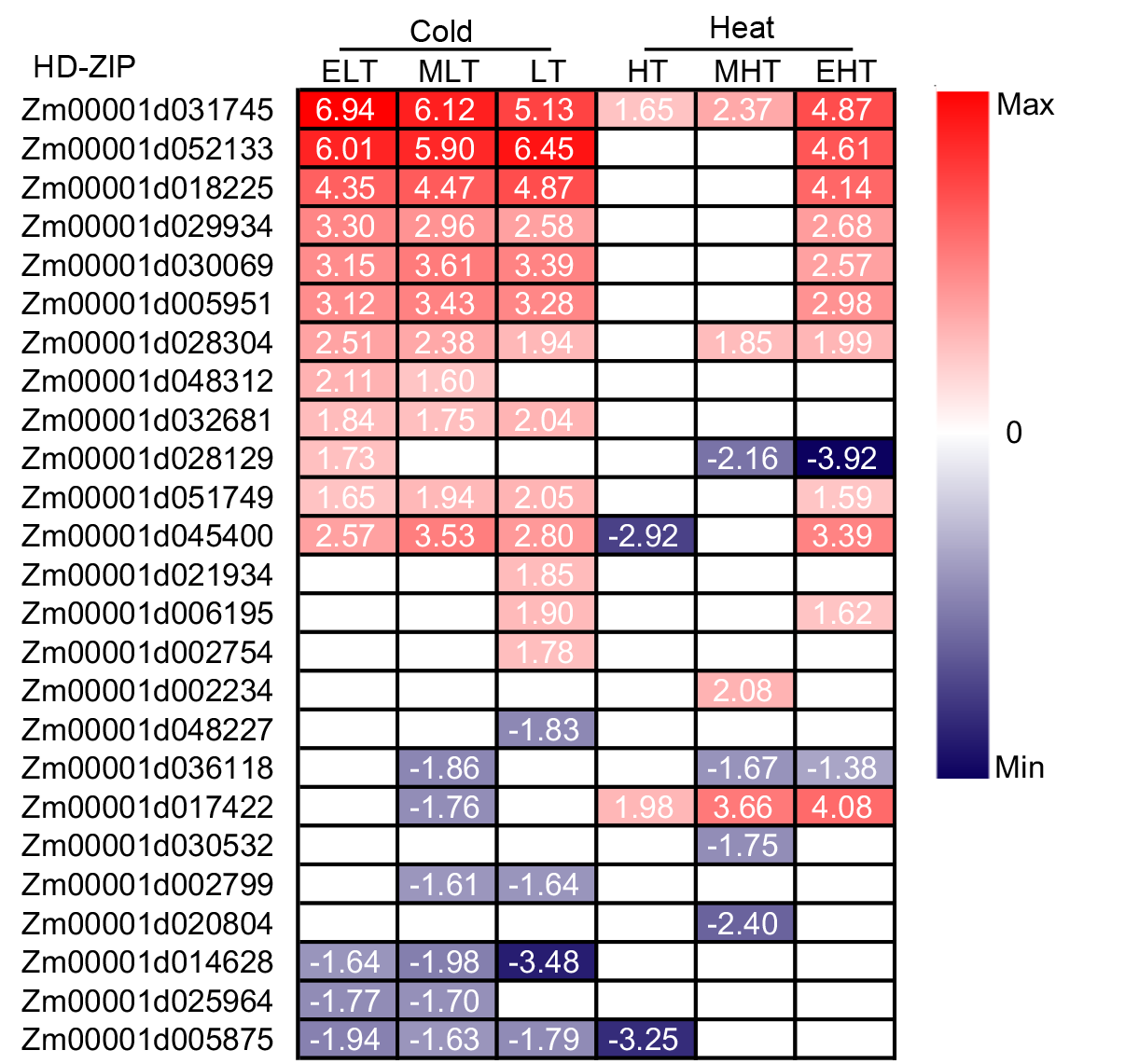

Supplement: Supplementary file 1 [file genes-11-00881-s001.zip › Supplemental Materials/Figure S4-HD-ZIP.tif]

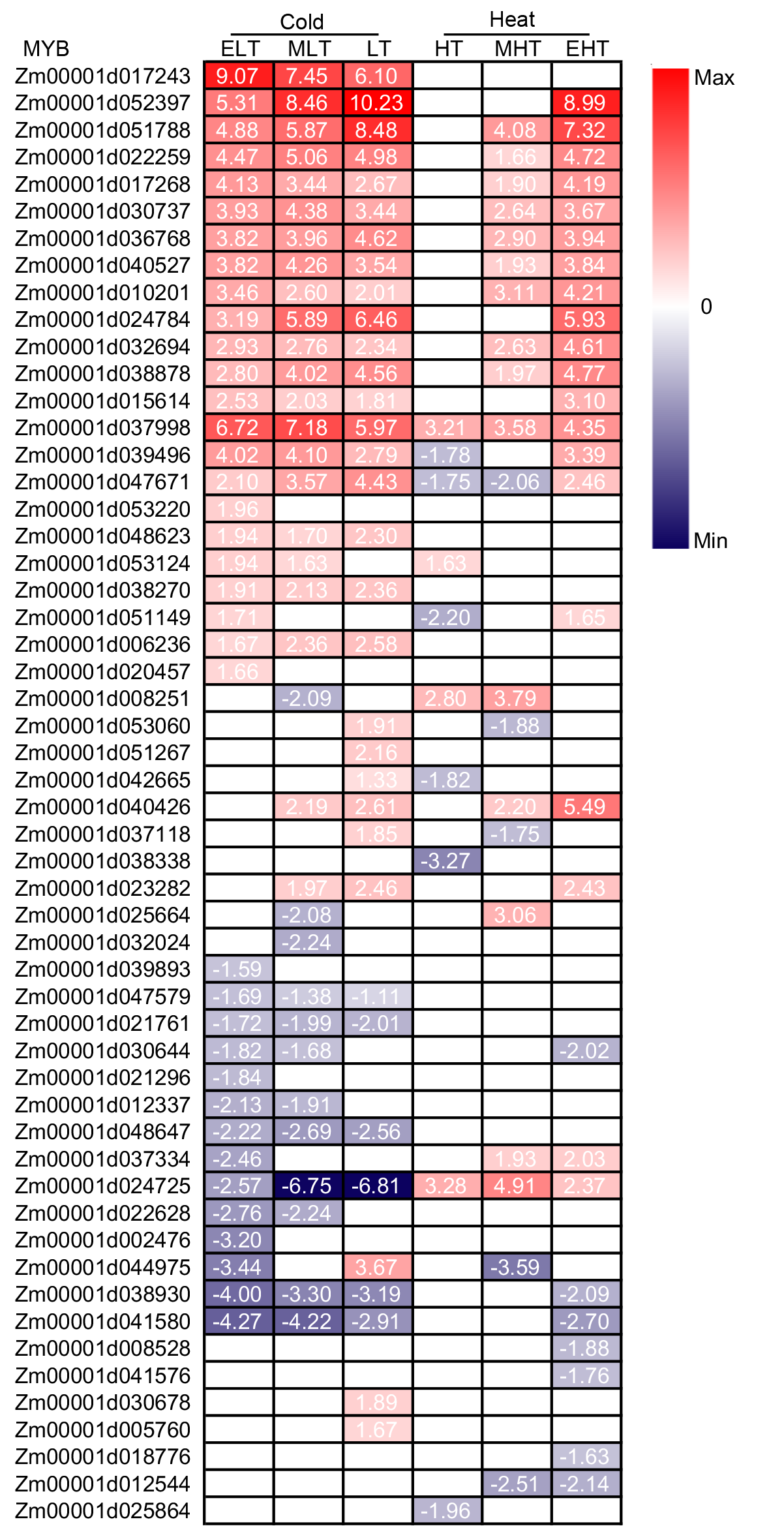

Supplement: Supplementary file 1 [file genes-11-00881-s001.zip › Supplemental Materials/Figure S5-MYB.tif]

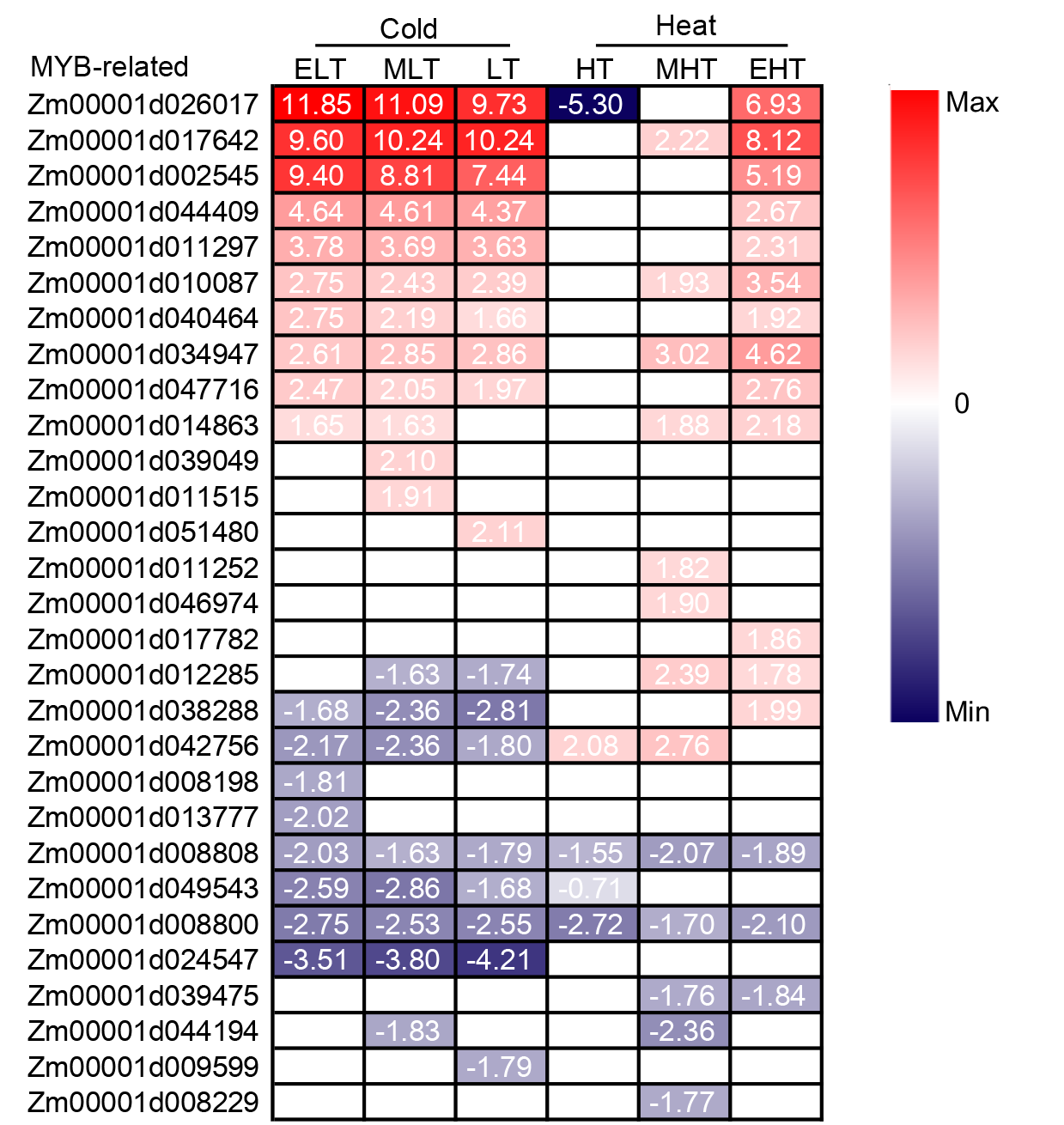

Supplement: Supplementary file 1 [file genes-11-00881-s001.zip › Supplemental Materials/Figure S6-MYB-related.tif]

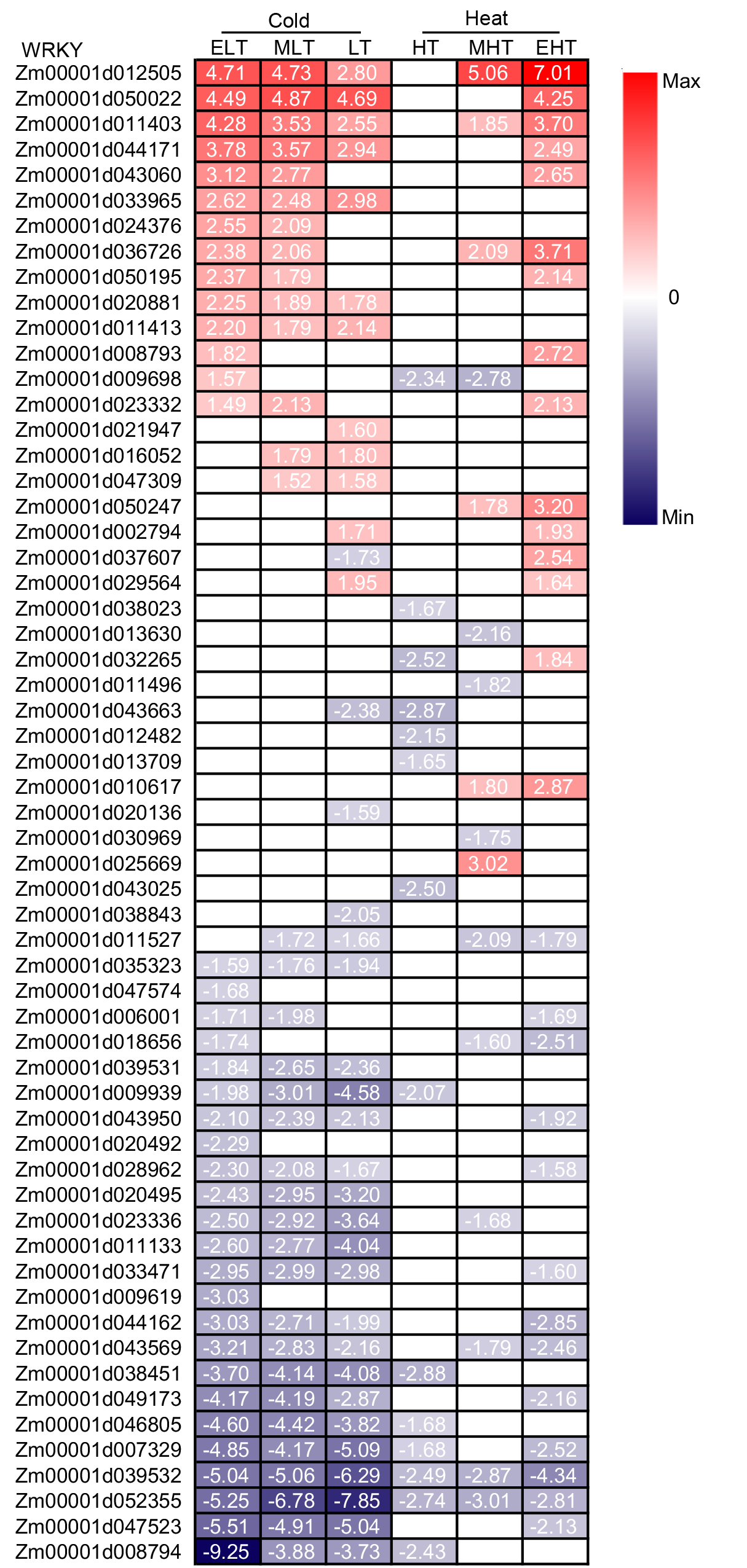

Supplement: Supplementary file 1 [file genes-11-00881-s001.zip › Supplemental Materials/Figure S7-WRKY.tif]

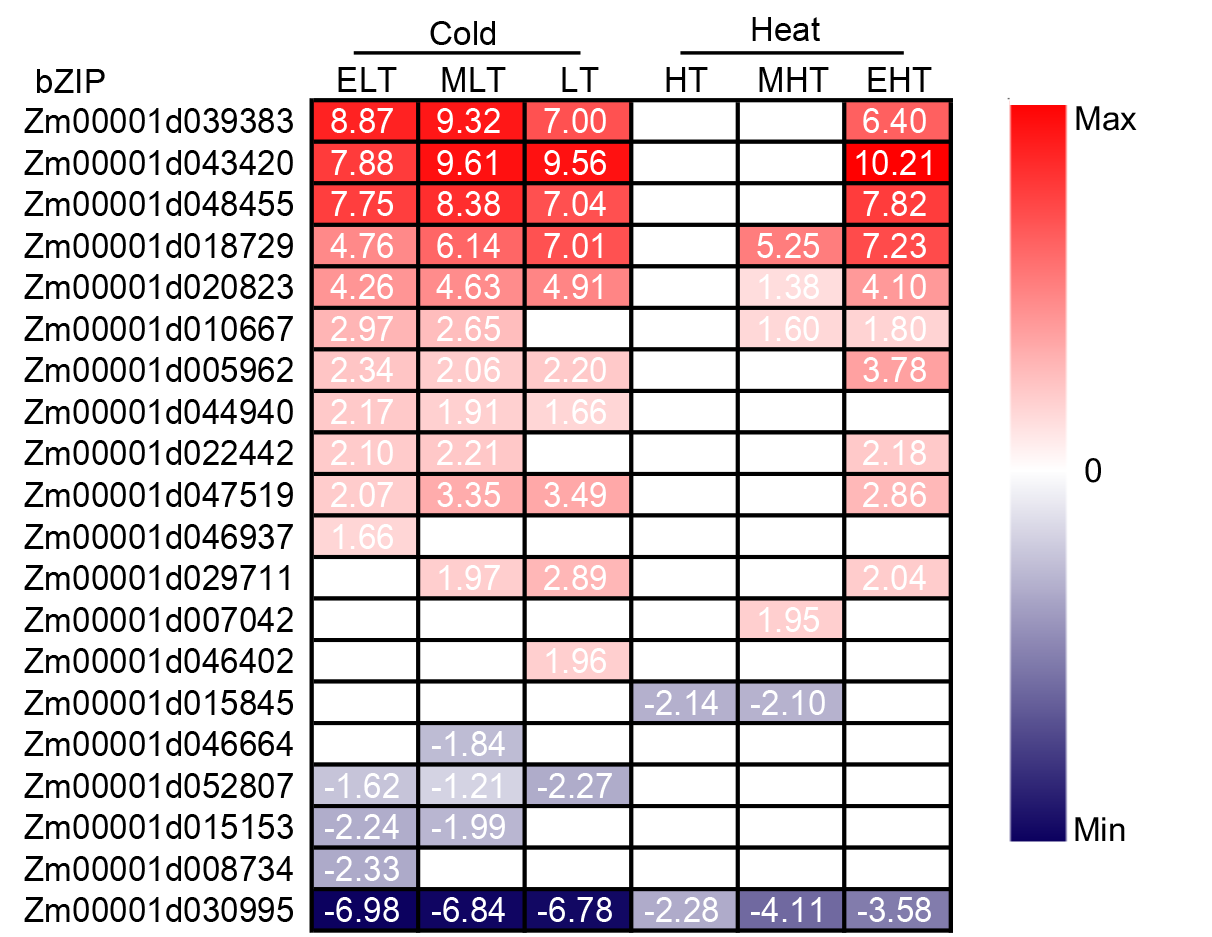

Supplement: Supplementary file 1 [file genes-11-00881-s001.zip › Supplemental Materials/Figure S8-bZIP.tif]

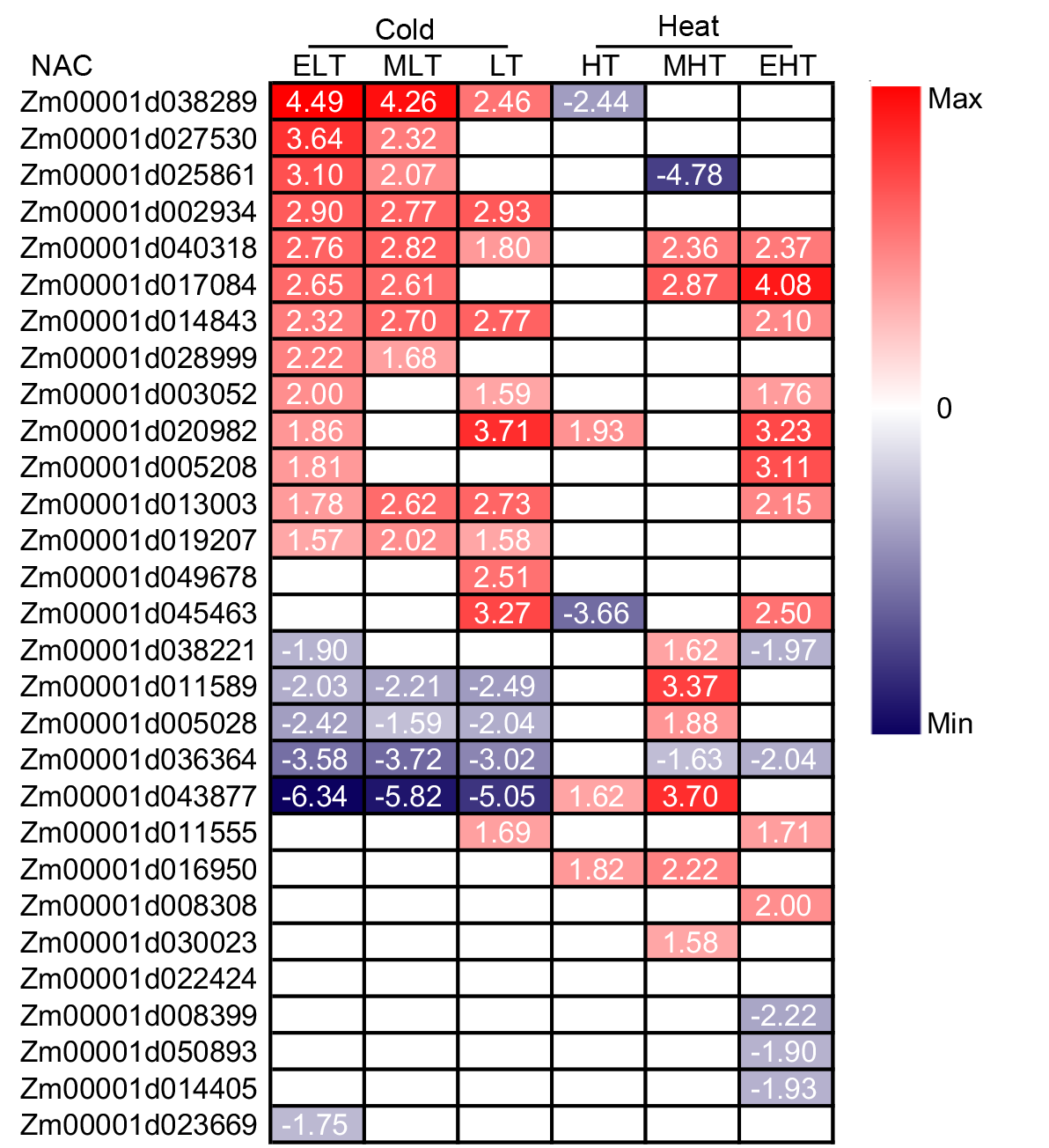

Supplement: Supplementary file 1 [file genes-11-00881-s001.zip › Supplemental Materials/Figure S9-NAC.tif]
